# Who were the Hyksos? Challenging traditional narratives using strontium isotope (^87^Sr/^86^Sr) analysis of human remains from ancient Egypt

**DOI:** 10.1371/journal.pone.0235414

**Published:** 2020-07-15

**Authors:** Chris Stantis, Arwa Kharobi, Nina Maaranen, Geoff M. Nowell, Manfred Bietak, Silvia Prell, Holger Schutkowski

**Affiliations:** 1 Department of Archaeology and Anthropology, Bournemouth University, Poole, United Kingdom; 2 PACEA - De la Préhistoire à l'Actuel: Culture, Environnement et Anthropologie, UMR CNRS 5199, Université de Bordeaux, Pessac cedex, France; 3 Department of Earth Sciences, Durham University, Durham, United Kingdom; 4 Austrian Academy of Sciences Vienna, Vienna, Austria; University at Buffalo - The State University of New York, UNITED STATES

## Abstract

A foreign dynasty, known as the Hyksos, ruled parts of Egypt between c. 1638–1530 BCE. Their origins are thought to be rooted in the Near East, which is supported by architectural features and grave accoutrements of Tell el-Dab^c^a. In this former Hyksos capital in the Eastern Nile Delta, burial culture is characterized by a blend of Egyptian and Near Eastern elements. However, investigations are still ongoing as to where the Hyksos came from and how they rose to power. The aim of this study is to elucidate the question of possible provenience. We present the results of strontium isotope (^87^Sr/^86^Sr) ratios of human tooth enamel (*n* = 75) from Tell el-Dab^c^a, focusing on comparing pre- and during Hyksos rule and sex-based differences. An influx of non-locals can be observed in the pre-Hyksos period (12^th^ and 13^th^ Dynasties, c. 1991–1649 BCE) during the constitution of this important harbor town, while the number of individuals already born in the Delta is larger during the Hyksos period. This is consistent with the supposition that, while the ruling class had Near Eastern origins, the Hyksos’ rise to power was not the result of an invasion, as popularly theorized, but an internal dominance and takeover of foreign elite. There is a preponderance of non-local females suggesting patrilocal residence. We discuss our findings against the current evidence of material culture and historiography, but more investigation in Near Eastern comparative sites has to be conducted to narrow our future search for the actual origins of the Hyksos.

## Introduction

The narrative of how the 15^th^ Dynasty of ancient Egypt (c. 1640–1530 BCE), known as that of the Hyksos, rose to rule is apocryphal. The Ptolemaic priest Manetho was for centuries the only account of their rise, rule, and fall [1, 2]. Living approximately twelve centuries after the Hyksos dynasty, Manetho described the Hyksos rulers as leading an invading force sweeping in from the northeast and conquering the northeastern Nile Delta during the Second Intermediate Period in a time when Egypt as a country was vulnerable [1]. Manetho’s account only survived in the works of later historians such as Flavius Josephus and, however biased and unreliable, was the solitary known source of the Hyksos for centuries [3–5].

Even after the decipherment of hieroglyphs, sources for the Hyksos rulers remained scarce and unreliable due to the ancient Egyptian stately customs of censorship and propaganda; Hyksos became a part of the *topoi* depicting disorder and chaos, whose ritual killing was the Pharaohs’ way to maintain order and legitimize power [6–8]. Western scholars further entangled the origins of the Hyksos with race-based ‘science’ mired in Imperialism and Orientalism, conflating the Hyksos rulers to represent an entire ethnic group that further confused pursuits to investigate the origins of the 15^th^ Dynasty rulers [9–13]. During this paper, we only refer to the dynastic rulers as the Hyksos, not the elite attendant to the rulers nor that ethnic group with which they are associated.

**Fig 1:**
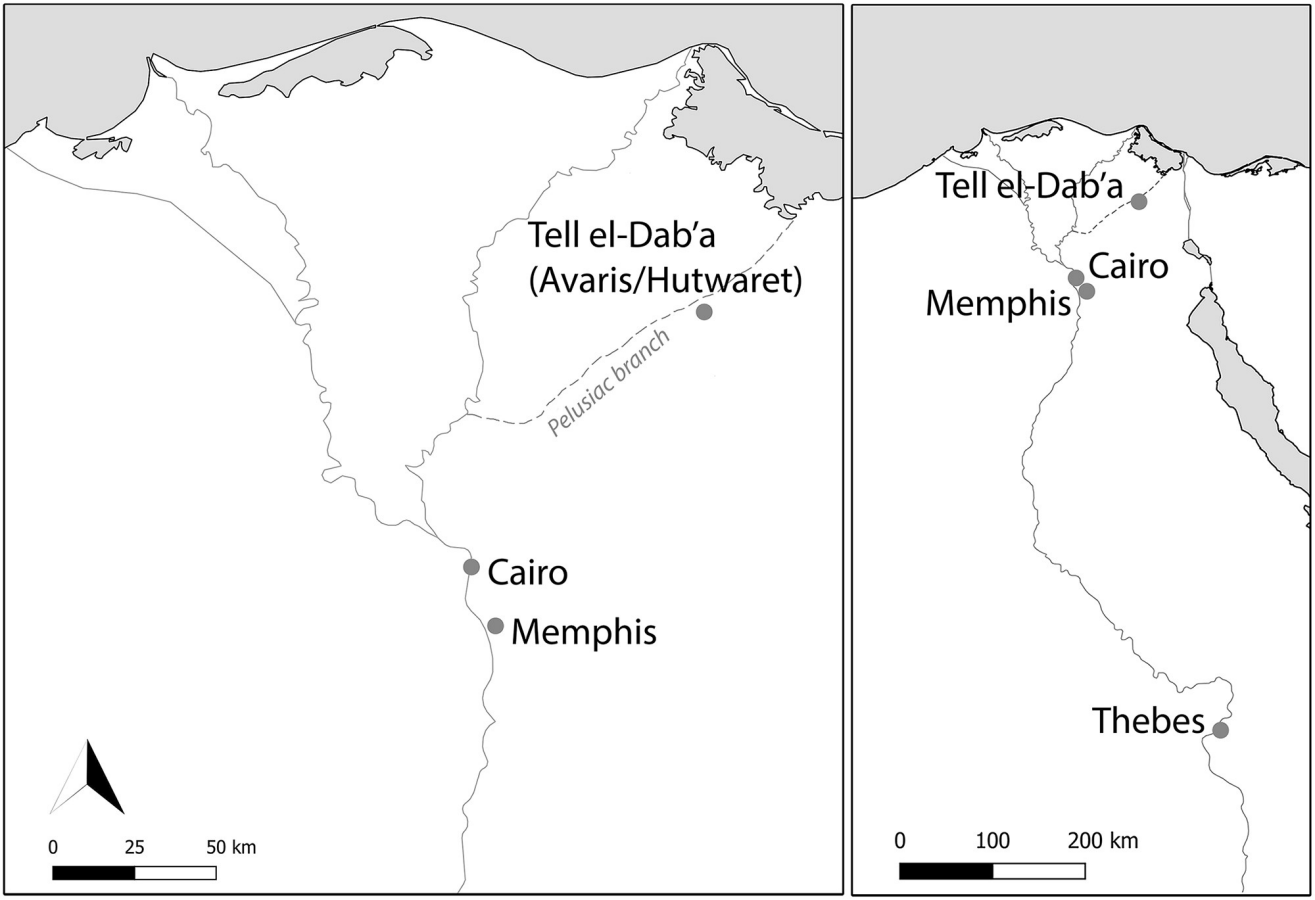
Location of Tell el-Dab^c^a in Egypt’s eastern Delta.

With the discovery of the ancient Hyksos capital at the archaeological site of Tell el-Dab^c^a (Fig 1) and five decades of excavation including several cemetery sites, an opportunity arises to investigate the circumstances in which the Hyksos rose to rule [14]. The last decades of research have produced evidence clearly pointing towards a Near Eastern origin of the ruling class known as the Hyksos, notably borne out by shared non-Egyptian features of ceramic types, burial customs, adornment, weapons, as well as domestic and cultic architecture, though not the foreign elite arriving directly from foreign lands as Manetho recounted but people of non-Egyptian ethnicity who were born and raised in the Delta [14–16]. To date, no tomb known to belong to a Hyksos ruler has been excavated, but this wealth of new material and insights allows direct comparison with the Levant and the wider Near East in a significant step forward towards explaining cultural trends and geographic provenance of people associated with the Hyksos and the background of their migration into the northeastern Nile Delta. The archaeological evidence also does not support Manetho’s narrative of the Hyksos as leading an invading force sweeping in from the northeast to rule as Egypt’s first foreign dynasty; instead, it is suggested that those who became Hyksos rulers were descended from Asiatics who had been living in Egypt for generations [15].

### The site of Tell el-Dab^c^a

**Fig 2:**
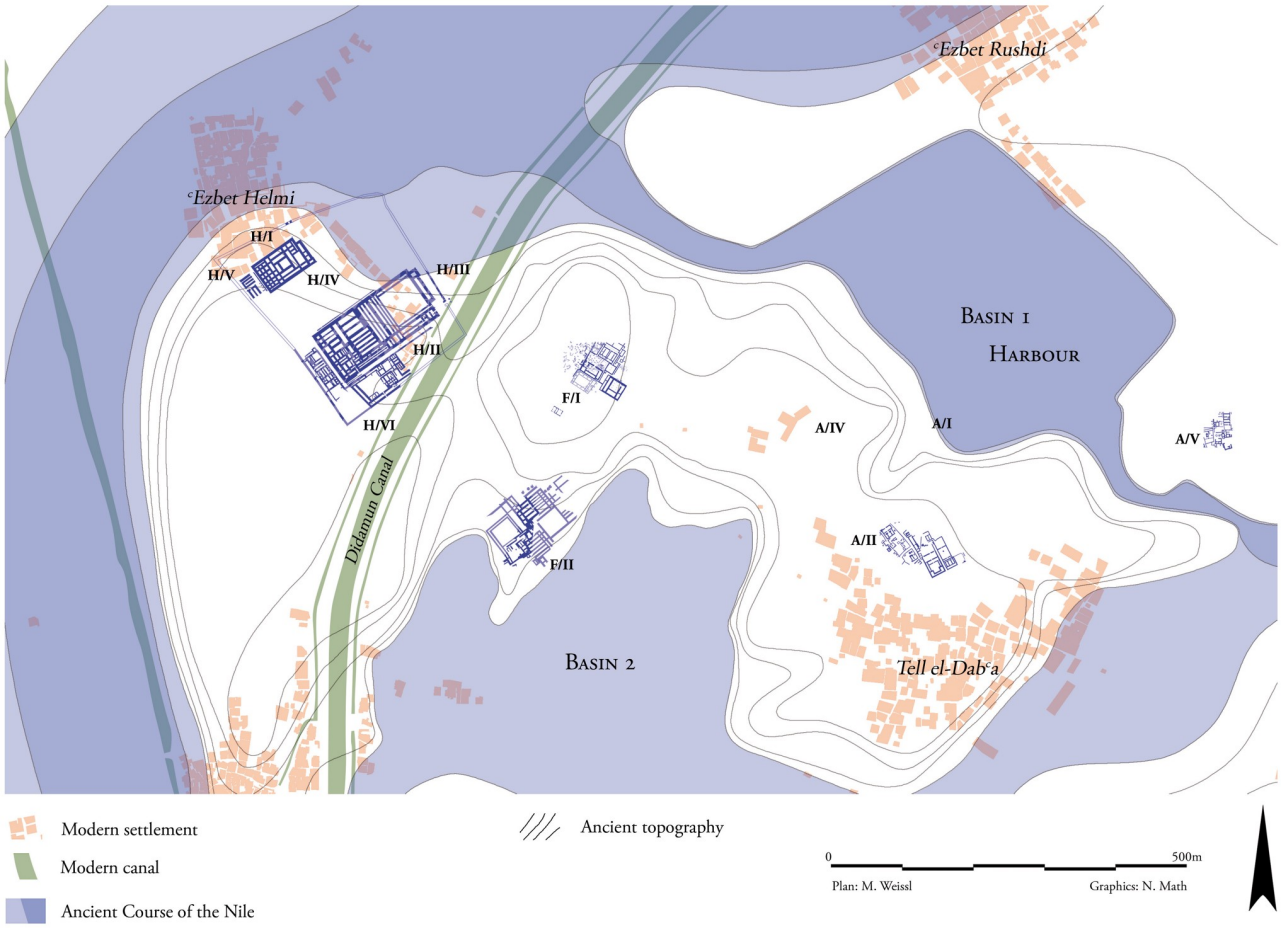
Site plan of Tell el-Dab^c^a and nearby sites of ʿEzbet Helmi and ʿEzbet Rushdi.

The examination of individuals buried in the cemeteries of Tell el-Dab^c^a offers the opportunity to directly assess the origins of these residents and assess questions relating to timing and mechanisms of the Hyksos’ rise to rule (for a brief history of excavation of the site, see [17] pp. 23–24). The site, located in the northeastern Nile Delta, has revealed a stratigraphy extending over 500 years [14, 15] (Fig 2). This settlement was founded in the 12^th^ dynasty and was known from the 13^th^ dynasty onwards as Hutwaret [18–20]. During the Middle Kingdom, this city was an administrative center and a harbor city that grew in power to finally become the capital of the regional Hyksos Kingdom. Then known as Avaris, various peoples and groups from both near and far negotiated their concepts of life, religion, technology, politics and power in this harbor hub [1, 21–25]. The city was later largely abandoned after around 1550 BCE, following the campaigns of the southern Theban Kingdom (the 17^th^ Dynasty) in its pursuit to defeat the Hyksos rulers and forge the New Kingdom [15, 26, 27], although a palace complex in the nearby site of ʿEzbet Helmi dating to the early 18^th^ dynasty shows a certain continuity within this area [28].

**Fig 3:**
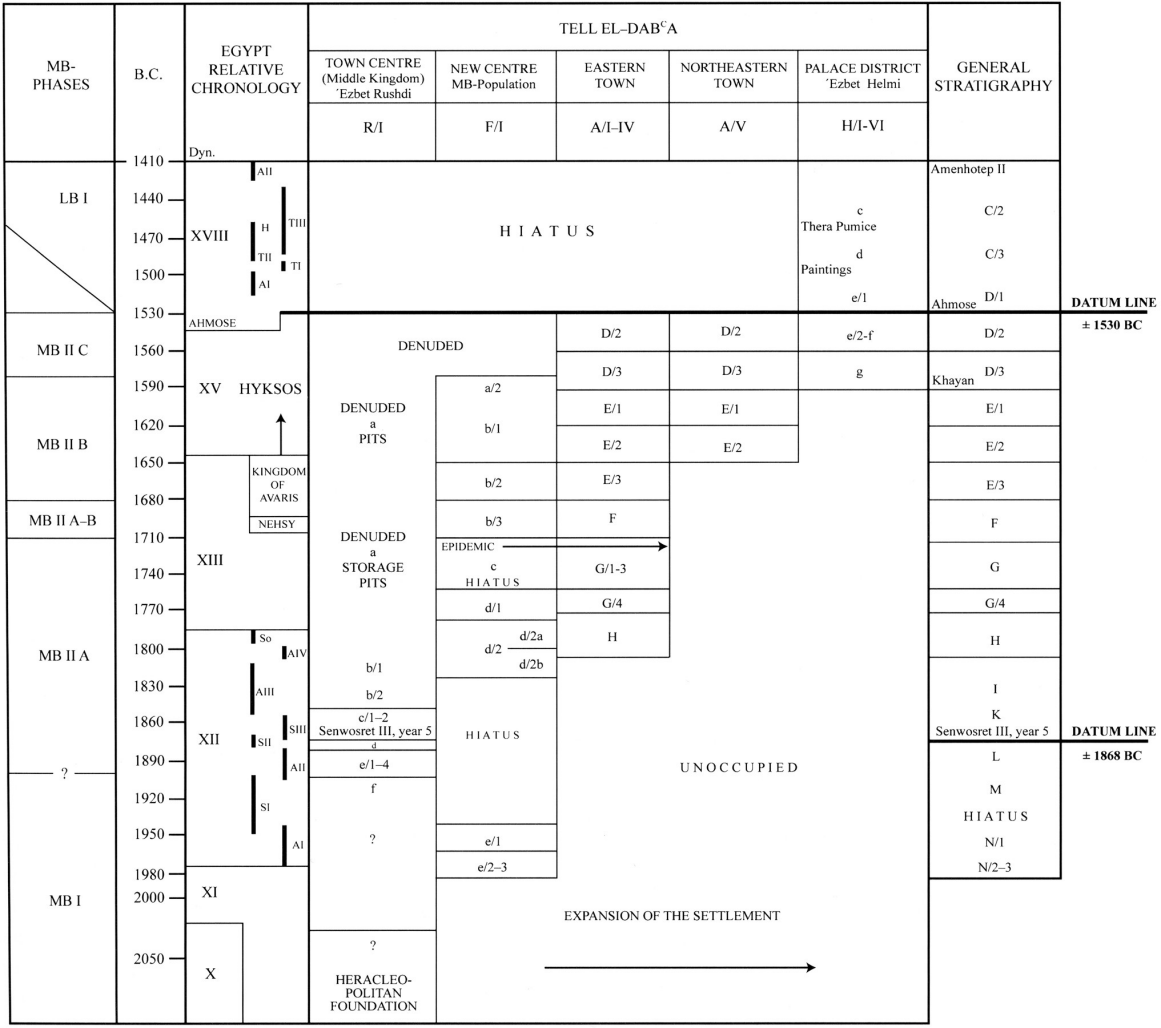
The site stratigraphy system. After [15].

Samples come from cemeteries in Areas A/I, A/II, and F/I as seen on Fig 2. Area A/I has only one individual for the current analysis available at the museums where the specimens were collected, and it is also the smallest (and least-published) of the three areas [29]. The Tell el-Dab^c^a stratigraphy system and how the strata compare with relative and estimated absolute chronologies can be seen on Fig 3. All metalwork from Area A/I and A/II comes after stratum E/1-D/3 during the Hyksos time period [13].

Area A/II is the largest cemetery of the site, as well as the largest sample in this study, and it is the most comprehensively published area of Tell el-Dab^c^a. Occupation in Area A/II began with small scale settlement activity throughout strata H-G/1 [30]. The appearance of a distinct eastern Delta material culture, interpreted as that of the Fifteenth Dynasty, was identified from stratum E/2-1 onwards, with further changes during D/2 [31]. Large temples were built in the Area during stratum F, E/3 and E/2. Temple III, built during stratum F and E/3, continued in use throughout the time period. Continuation in land ownership (visible in unchanging land plots) and the use of Temple III, has been considered indicative of wider continuation spanning from before the Hyksos period [31, 32].

Area F/I stratigraphy is marked by lower case letters, differing from the general site stratigraphy that is indicated by capital letters. The earliest occupation in Area F/I dates to the 12^th^ Dynasty, strata e/2-3 [33]. The following stratum d/2 has examples of non-local customs, ranging from domestic architecture, pottery and burials to Near Eastern style metalwork [13]. Area F/I exhibits a range of burial structures during strata d/1-2 that have not been found outside this area or time period. Afterwards, burial structures unify and resemble those of the rest of the site [34].

### The research questions

As the cemeteries in Tell el-Dab^c^a hold individuals interred before and during Hyksos rule [31, 34, 35], this paper addresses three questions related to the influx/movement of the people interred at Tell el-Dab’a:

1. Is there a chronological pattern to the movement? I.e., is there markedly more influx into Tell el-Dab^c^a during the 12^th^ Dynasty or during the 15^th^ Dynasty?
2. Are there any sex-based patterns in movement?
3. Can non-locals be confidently provenanced?

Questions One and Two address the issue surrounding the Hyksos origin and mechanisms of rise to rule. An influx of foreigners during Hyksos rule at the city that would become the Hyksos capital would lend some strength to the idea of an invading force. A larger influx of male non-locals would also lend support to this concept. Stable economic power might open up opportunities for whole families to move into the Nile Delta and settle; in this instance, gender parity in non-locals might be expected. Question Three investigates the potential origin of the non-locals at Tell el-Dab^c^a, to address what is perhaps the greatest question around the 15^th^ Dynasty: where did the Hyksos come from?

### Strontium isotope (^87^Sr/^86^Sr) analysis in Egypt

Strontium isotope (^87^Sr/^86^Sr) analysis of human tissues provides insight into residential mobility and origin on the individual level, allowing extrapolations into largescale socio-political dynamics [36–40]. Multiple paleomobility studies have utilized ^87^Sr/^86^Sr on human dental enamel to identify non-locals in Egyptian and Sudanese contexts along the Nile Valley [41–45], although none have been conducted on humans from the Nile Delta region. Interpretation of strontium isotope analysis rests on the assumption that an individual’s body tissues will reflect the ^87^Sr/^86^Sr values of the underlying geology in which they lived when these tissues were forming, with no appreciable fractionation across trophic levels or in metabolic processes [46]. Recent research suggests that fertilization with lime in modern agriculture affects interpretation of strontium isotopes [47], although that is not expected to be a major issue in the fertile Nile Delta.

The Nile River is formed from the Blue Nile tributary running through modern-day Ethiopia and Sudan and the White Nile tributary, which, depending on the definition, either begins at Lake No in South Sudan or further north in the African Great Lakes Region [48]. The southern geological formations are heterogeneous and complex, but erosion of these formations by the Nile create low variability of biogenically available ^87^Sr/^86^Sr values when the river reaches the Delta.

Although plant material is generally accepted to be ideal for forming a bioavailable strontium baseline [49–51], exportation of modern botanical samples were not within the purview of this study. Instead, previous ^87^Sr/^86^Sr analysis of Tell el-Dab^c^a animal bones was used as proxy for the local biosphere [52]. These animal samples have demonstrated that the local region has a restricted bioavailable ^87^Sr/^86^Sr range. This restricted range of local values is ideal for differentiating between those who spent their childhood in the northeastern Nile Delta and those who grew up somewhere else and then moved to the region. The caveat is that the wider Nile Valley also has a fairly restricted range similar to the local range, and so identifying non-locals who originated from this region might be impossible using strontium isotope analysis alone.

## Materials and methods

During excavations at Tell el-Dab^c^a in the late sixties and early seventies, archaeological samples were exported after a find partition in The Museum of Egyptian Antiquities (Cairo, Egypt) to Vienna, Austria. The majority of the cemetery assemblage was stored in the Anthropological Department of the Natural History Museum of Vienna, the Anthropological Department of the University of Vienna and the Medical University of Vienna. Permission from each of these curation institutions was secured in order to sample, export and perform destructive analysis. No further permits were required for the described study, which complied with all relevant regulations.

A site chronology has been established by timelines provided by a stela of Sesostris III and the abandonment of the site at the end of the Hyksos Period along with comparative ceramic studies with well-dated sites in Egypt and further refined with ^14^C dating [53, 54]. In order to address the question of chronological patterns of movement, we collapse the detailed timescale to a simple ‘Pre-Hyksos’ time period for the site encapsulating the 12^th^ and 13^th^ Dynasties (1991–1649 BCE) and the Hyksos reign. Age and sex estimations were determined using standard bioarchaeological methods [55, 56], with Winkler and Wilfing's observations as field osteologists at the site used as supplementary information [57].

Second permanent molars or permanent premolars (first or second) were selected preferentially as these teeth, whether mandibular or maxillary, complete crown formation between three and eight years of age [58]. Third molars could be selected if the second molars or premolars were damaged, missing, or otherwise unavailable: in some instances the preferred teeth were ‘cemented’ to the alveolar and were not selected to reduce damage to the collection. First molars were less ideal as their more precocious development causes some maternal influence during the *in utero* development of the crown [58], but were selected when no other molar or premolar was available.

With these sampling limitations, 75 individuals were available to analyze. Of those, 30 are estimated to be female and 20 are estimated to be male. Regarding chronology, all but one (A/II-m/11 Westprofil) came from secure contexts with assigned site phases, although three came from site phases intermediate to the pre- and during- Hyksos periods and could not be assigned using this dichotomous system; 36 are considered ‘pre-Hyskos’ and 35 are from the time of Hyksos rule. The individuals sampled were excavated from three areas of the site: A/I (*n* = 1), A/II (*n* = 67), and F/I (*n* = 7). We will need to first test whether the two areas with larger sample sizes are displaying significantly different ^87^Sr/^86^Sr values as groups before combining to test for sex and time period.

### Analytical methods

Initial sample preparation was conducted in the Department of Archaeology and Anthropology Dorset House laboratory at Bournemouth University. The crown of each tooth was sandblasted to remove the outer layer and surface contaminants. A small piece of enamel was cut from the tooth using a dental rotary tool. Any dentine attached to the sample was ablated with a diamond-tipped engraving cutter. After being sonicated rinsed three times with MilliQ water, samples were purified using the ion exchange method presented outlined by previous research [59, 60]. The cleaned enamel samples were weighed, dissolved in concentrated HNO_3_, and then diluted to 3M HNO_3_. The sample solutions were then loaded onto columns with Eichrom Sr-spec resin, eluted with MilliQ water, and acidified to 3% HNO_3_ for analysis.

Strontium isotope ratios were measured using a ThermoFinnigan Multi-collector ICP Mass Spectrometer (MC-ICP-MS) in the Department of Earth Sciences at Durham University (United Kingdom). Reproducibility of the standard NBS987 during sample analysis was 0.710254 ± 0.000006 (2SD, *n* = 59). All NBS987 values have been normalized to the accepted value of 0.710240 [61, 62].

An individual is considered ‘local’ if their ^87^Sr/^86^Sr value falls within the animal baseline mean value ± two standard deviations (0.70761–0.70780) [52]. This generates wider limits than the total range of animal ^87^Sr/^86^Sr values (0.70766–0.70778), creating more conservative parameters for ‘local’ values. Statistics were conducted using the open-source software R [63].

## Results

Demographic, sampling, and isotopic data are presented on S1 Table, with a summary of the results shown on Table 1.

**Table 1:** Summary of ^87^Sr/^86^Sr results for the site of Tell el-Dab^c^a.

	Mean	SD	*n*
**Total**	0.70790	0.00023	75
**Sex**			
***Female***	0.70801	0.00029	30
***Male***	0.70786	0.00021	20
**Time Period**			
***Pre-Hyksos***	0.70797	0.00025	36
***Hyksos***	0.70783	0.00021	35

**Fig 4:**
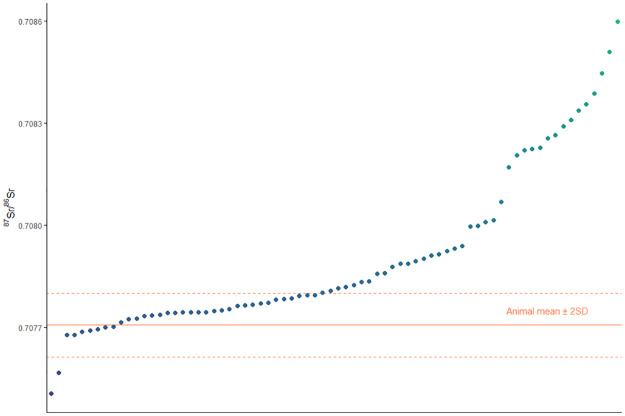
^87^Sr/^86^Sr values of all individuals from the archaeological site of Tell el-Dab^c^a.

**Fig 5:**
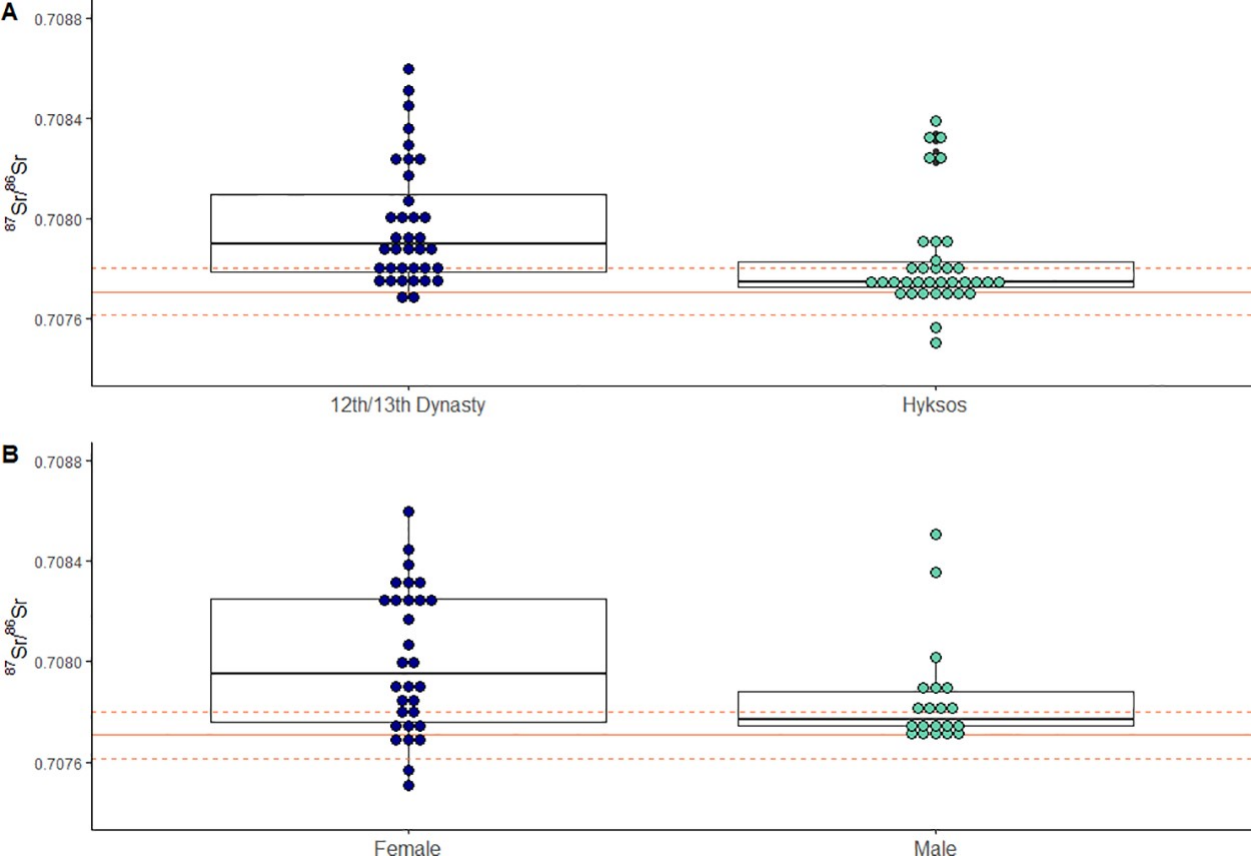
Comparing ^87^Sr/^86^Sr values by time period and sex. (A) Comparison of ^87^Sr/^86^Sr values of pre-Hyksos and Hyksos individuals. (B) Comparison of ^87^Sr/^86^Sr values of females and males.

**Fig 6:**
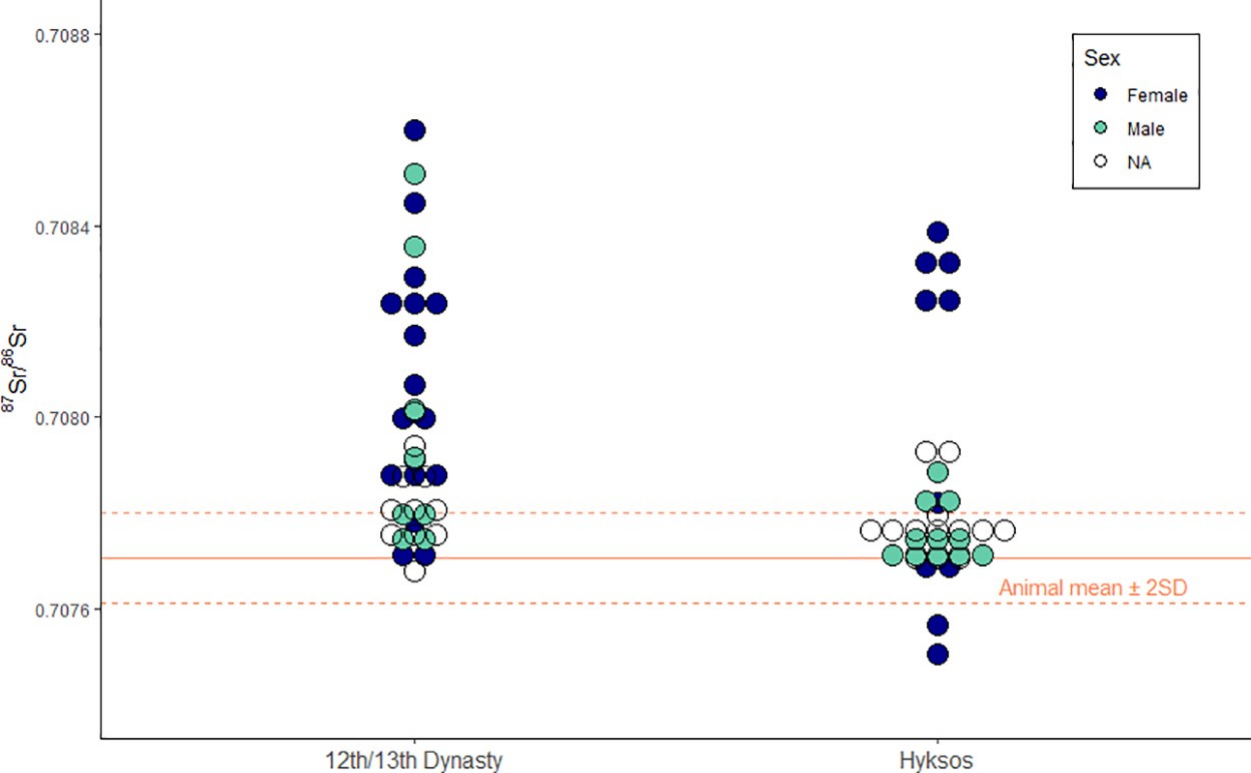
Dot plot showing ^87^Sr/^86^Sr results by time period, with the dots colored by sex.

When plotted against the local strontium values, more than half of all individuals (40/75 or 53%) from Tell el-Dab^c^a spent their childhood outside the Nile Delta (Fig 4). There are no significant differences between the excavation areas (t(74) = -1.24, *p* = 0.250), and so they are combined to compare time periods and the sexes. Those individuals outside the local range display a wide range of values both above and below the Nile Delta range. There were significant differences in the ^87^Sr/^86^Sr values for Pre-Hyksos and Hyksos individuals; t(70) = 2.6, *p* = 0.012, with more immigrants during the Pre-Hyksos time periods (Fig 5A). There were also significant differences between the sexes; t(49) = 2.1, *p* = 0.041, where more females are non-locals compared to males (Fig 5B). Breaking down the sub-groups by sex and time period creates small sub-groups that are not ideal for statistical testing, but visual examination of the patterns are available on Fig 6. When plotting isotope values by both time period and sex, we see some clusters of non-local women from the Hyksos period: five with higher-than-local values and two with the lowest values in the assemblage.

## Discussion

Given that the Nile River is the main water source for drinking and watering crops through ancient Nubia and Egypt, the strontium values for the majority of these ancient populations would fit close to the local range of Tell el-Dab^c^a. Individuals who grew up along the Nile, where alluvium creates a corridor of homogenous ^87^Sr/^86^Sr values, likely show values too similar to differentiate between, for example, Upper and Lower Egypt [52]. As such, non-locals south of the northeastern Nile Delta would show the same strontium values as locals to the region of study; individuals from major centers such as Memphis, Thebes, and even further south into Upper Egypt and Nubia might be present in this assemblage but unidentifiable using strontium isotope analysis.

The Hyksos-era women clustering higher and lower than the local biospheric values on Fig 4 are interred at different tombs across the site. It is possible that women hailed from the same origins in this time, but this might be an issue of equifinality and they have childhood residences in different parts of the world with similar underlying geologies [64–66]. Oxygen stable isotope (δ^18^O) analysis is an additional tool for investigating paleomobility in a bioarchaeological context [67] and has been used in previous Egyptian and Sudanese studies [45, 68–72]. Future research utilizing δ^18^O analysis on the Tell el-Dab’a assemblage might hold promise for identifying these non-locals with local ^87^Sr/^86^Sr values.

Despite a reasonable expectation of isotopic homogeneity, the majority of individuals in the larger assemblage irrespective of time period show non-local ^87^Sr/^86^Sr values, which is compelling. Chronological patterns of movement can be observed using ^87^Sr/^86^Sr analysis on human remains from the site of Tell el-Dab^c^a, with more immigrants previous to the Hyksos Dynasty. On a local scale, this reflects in some way the international characteristic of the city as a harbor in the northeastern Nile Delta. In combination with previous archaeological evidence [15], this research supports the concept that the Hyksos were not an invading force occupying this city and the upper Nile Delta, but an internal group of people who gained power in a system with which they were already familiar.

Contrary to the model of the Hyksos coming to power from a foreign invasion, we did not find more males moving into the region. Gender parity would have been expected with families moving as economic opportunities arose, but instead we find a sex bias towards females. The greater proportion of non-local females compared to males could fit with patrilocality in Egypt and the Near East [73], but this rather high proportion of 77% of females as non-local deserves more careful contextual consideration.

The excavated cemeteries and domestic burials are assumed to be more representative of the elites of the city rather than the ‘common’ population [13], and it is possible that these women are coming to the region for marriages cementing alliances with powerful families from beyond the Nile. During the Middle Kingdom and Second Intermediate Period, there is more documentation of men with Egyptian names marrying women with non-Egyptian names than vice versa [74]. This attitude towards marriage to foreign families continues into the 18^th^ Dynasty [75]: foreign women could marry into high status Egyptian families, but Egyptian women would not marry foreign kings. It would be interesting if the technological and cultural transmission of the Hyksos dynasty on later Egyptian culture could be viewed through the lens of gender theory to explore this potential contribution from the influx of immigrant women, if the collection analyzed in this paper is indeed representative of the larger migration patterns.

## Conclusions

Isotope analysis is a powerful tool for exploring past mobility and identifying non-locals. However, exploring the origin of non-locals using this method is much more difficult. The wide range of values in the Tell el-Dab^c^a assemblage suggests that non-locals, either before or during Hyksos rule, did not come from one unified homeland, but an extensive variety of origins. This in itself is interesting, as although those who would become Hyksos rulers might have an ancestral connection with a single homeland, the northeastern Nile Delta represented a multicultural hub long before the Hyksos rule.

Utilizing the extensive burial areas to contribute one of the largest isotopic studies of ancient Egypt to date, this study is the first to use archaeological chemistry to directly address the origins of the enigmatic Hyksos Dynasty, the first instance in which Egypt is ruled by those of foreign origin. Although the Levantine origin of these rulers is not in question due to their rulers’ names, architecture, and material culture, these results challenge the classic narrative of the Hyksos as an invading force. Instead, this research supports the theory that the Hyksos rulers were not from a unified place of origin, but Western Asiatics whose ancestors moved into Egypt during the Middle Kingdom, lived there for centuries, and then rose to rule the north of Egypt.

## Supporting information

S1 Table Demographic information, relative dating, and isotopic information for each sample. For sex estimation, F = Female, M = Male, I = Indeterminate. For curation location, NHM = Anthropological Department of the Natural History Museum of Vienna, UV = the Anthropological Department of the University of Vienna and MUV = the Medical University of Vienna.
(DOCX)
