# Supplementary material for: Who were the Hyksos? Challenging traditional narratives using strontium isotope (87Sr/86Sr) analysis of human remains from ancient Egypt
Source: PLoS One. 2020 Jul 15;15(7):e0235414. doi: 10.1371/journal.pone.0235414 (PMC7363063; doi:10.1371/journal.pone.0235414)
Supplement: S1 Table — For sex estimation, F = Female, M = Male, I = Indeterminate. For curation location, NHM = Anthropological Department of the Natural History Museum of Vienna, UV = the Anthropological Department of the University of Vienna and MUV = the Medical University of Vienna. (DOCX) [file pone.0235414.s001.docx]

**S1 Table.** **Demographic, relative dating, and isotopic information for each sample.** For sex estimation, F = Female, M = Male, I = Indeterminate. For curation location, NHM = Anthropological Department of the Natural History Museum of Vienna, UV = the Anthropological Department of the University of Vienna and MUV = the Medical University of Vienna.

| **Burial Designation** | **Project Sample ID** | **^87^Sr/^86^Sr** | **Sr SE** | **Tooth Sampled (FDI)** | **Sex** | **Age category** | **Site Phase** | **Simplified Phase System** | **Curation Location** |
| --- | --- | --- | --- | --- | --- | --- | --- | --- | --- |
| A/I-g/3 Grab 1, Bestattung 1 | 183 | 0.707724 | 0.00001 | 28 | --- | Young Adult | E/1-D/3 | Hyksos | UV |
| A/II-k/12 Grab 1 | 544 | 0.708509 | 0.000008 | 47 | M | Young Adult | G-F | 12th/13th Dynasty | NHM |
| A/II-k/12 Grab 1 (2) | 545 | 0.708356 | 0.000006 | 47 | M | Middle Adult | G-F | 12th/13th Dynasty | NHM |
| A/II-l/11 Grab 1, Bestattung 2 | 185 | 0.707735 | 0.000008 | 17 | --- | Late Childhood | G-F | 12th/13th Dynasty | UV |
| A/II-l/11 Grab 3, Bestattung 1 | 186 | 0.707901 | 0.000006 | 37 | F | Young Adult | F | 12th/13th Dynasty | UV |
| A/II-l/12 Grab 4 | 187 | 0.707782 | 0.000007 | 37 | F | Young Adult | G-F | 12th/13th Dynasty | UV |
| A/II-l/12 Grab 5, Bestattung 1 | 188 | 0.707744 | 0.000006 | 47 | M | Young Adult | F | 12th/13th Dynasty | UV |
| A/II-l/12 Grab 5, Bestattung 2 | 189 | 0.707915 | 0.000007 | 17 | M | Old Adult | F | 12th/13th Dynasty | UV |
| A/II-l/12 Grab 5, Bestattung 3 | 190 | 0.707793 | 0.000008 | 17 | --- | Adolescent | F | 12th/13th Dynasty | UV |
| A/II-l/14 Grab 7, Bestattung 1 | 191 | 0.707747 | 0.00001 | 47 | --- | Adolescent | E/3 | 12th/13th Dynasty | UV |
| A/II-l/14 Grab 7, Bestattung 4 | 192 | 0.707734 | 0.000008 | 27 | F | Young Adult | E/3 | 12th/13th Dynasty | UV |
| A/II-l/15 Grab 5 | 193 | 0.707886 | 0.000008 | 47 | F | Young Adult | F | 12th/13th Dynasty | UV |
| A/II-l/16 Grab 4, Bestattung 1 | 194 | 0.707792 | 0.000008 | 37 | M | Young Adult | F | 12th/13th Dynasty | UV |
| A/II-l/17 Grab 16 | 199 | 0.707505 | 0.000012 | 47 | F | Middle/ Old Adult | E/2 | Hyksos | UV |
| A/II-l/17 Grab 5, Bestattung 1 | 196 | 0.707762 | 0.000007 | 47 | --- | Late Childhood | D/3 | Hyksos | UV |
| A/II-l/17 Grab 5, Bestattung 2 | 197 | 0.707687 | 0.00001 | 47 | --- | Young Adult | D/3 | Hyksos | UV |
| A/II-l/17 Grab 5, Bestattung 3 | 198 | 0.707742 | 0.00001 | 37 | --- | Adolescent | D/3 | Hyksos | UV |
| A/II-m/10 Grab 2 Bestattung 2 | 283 | 0.707785 | 0.000007 | 17 | --- | Subadult | D/3 | Hyksos | UV |
| A/II-m/10 Grab 8, Bestattung 1 | 201 | 0.707803 | 0.000007 | 17 | M | Young Adult | F | 12th/13th Dynasty | NHM |
| A/II-m/11 Westprofil | 204 | 0.707834 | 0.00001 | 37 | F | Young Adult | --- | --- | UV |
| A/II-m/12 Grab 13, Bestattung 1 | 284.1 | 0.708265 | 0.00001 | 37 | F | Young Adult | E/2 | Hyksos | NHM |
| A/II-m/12 Grab 14 | 207 | 0.707744 | 0.000008 | 37 | M | Middle Adult | E/2-1 | Hyksos | UV |
| A/II-m/13 Grab 13, Bestattung 2 | 211 | 0.708256 | 0.00001 | 17 | F | Young Adult | E/3 | 12th/13th Dynasty | UV |
| A/II-m/13 Grab 6 | 209 | 0.708224 | 0.000008 | 27 | F | Young Adult | E/1 | Hyksos | UV |
| A/II-m/15 Grab 11 | 216 | 0.707996 | 0.000008 | 47 | F | Young Adult | G | 12th/13th Dynasty | UV |
| A/II-m/15 Grab 12, Bestattung 2 | 217 | 0.708291 | 0.000008 | 47 | F | Young Adult | E/3 | 12th/13th Dynasty | UV |
| A/II-m/15 Grab 8 | 213 | 0.70817 | 0.000007 | 47 | F | Young Adult | F | 12th/13th Dynasty | UV |
| A/II-m/15 Grab 9, Bestattung 2 | 258 | 0.707772 | 0.000008 | 47 | --- | Late Childhood | G | 12th/13th Dynasty | MUV |
| A/II-m/16 Grab 2, Bestattung 1 | 286 | 0.707745 | 0.00001 | 37 | M | Middle Adult | F | 12th/13th Dynasty | UV |
| A/II-m/16 Grab 3, Bestattung 2[1] | 219 | 0.707859 | 0.000007 | 27 | --- | --- | F | 12th/13th Dynasty | UV |
| A/II-m/16 Grab 3, Bestattung 3 | 302 | 0.707911 | 0.00001 | 34 | F | Young Adult | F | 12th/13th Dynasty | NHM |
| A/II-m/16 Grab 3, Bestattung 4 | 221 | 0.708221 | 0.00001 | 45 | F | Adolescent | F | 12th/13th Dynasty | UV |
| A/II-m/17 Grab 1, Bestattung 1 | 222 | 0.707766 | 0.000008 | 37 | --- | Late Childhood | D/3-2 | Hyksos | UV |
| A/II-m/17 Grab 3, Bestattung 10 | 230 | 0.707742 | 0.000007 | 47 | --- | Late Childhood | D/2 | Hyksos | UV |
| A/II-m/17 Grab 3, Bestattung 11 | 303 | 0.707699 | 0.000012 | 26 | --- | Young Adult | D/2 | Hyksos | UV |
| A/II-m/17 Grab 3, Bestattung 14 | 231 | 0.707702 | 0.000008 | 17 | F | Young Adult | D/2 | Hyksos | UV |
| A/II-m/17 Grab 3, Bestattung 2 | 333 | 0.707737 | 0.000006 | 37 | M? | Middle/ Old Adult | D/2 | Hyksos | UV |
| A/II-m/17 Grab 3, Bestattung 3 | 224 | 0.707737 | 0.00001 | 47 | M | Middle Adult | D/2 | Hyksos | UV |
| A/II-m/17 Grab 3, Bestattung 4 | 225 | 0.707725 | 0.000008 | 27 | M | Young Adult | D/2 | Hyksos | UV |
| A/II-m/17 Grab 3, Bestattung 5 | 226 | 0.707714 | 0.00001 | 45 | M | Middle Adult | D/2 | Hyksos | UV |
| A/II-m/17 Grab 3, Bestattung 6 | 227 | 0.707677 | 0.000008 | 17 | F | Adolescent | D/2 | Hyksos | UV |
| A/II-m/17 Grab 3, Bestattung 7 | 228 | 0.707691 | 0.000009 | 47 | M | Young Adult | D/2 | Hyksos | UV |
| A/II-m/17 Grab 3, Bestattung 8 | 229 | 0.707835 | 0.000008 | 37 | M | Young Adult | D/2 | Hyksos | UV |
| A/II-m/17 Grab 5 | 232 | 0.707878 | 0.000014 | 37 | M | Young Adult | E/3-2 | --- | UV |
| A/II-m/17 Grab 7 | 332 | 0.707765 | 0.000008 | 14 | --- | Middle Adult | E/3-2 | --- | UV |
| A/II-n/12 Grab 6 | 543 | 0.708598 | 0.000006 | lower premolar | F? | Middle Adult | F | 12th/13th Dynasty | NHM |
| A/II-n/13 Grab 1, Bestattung 1 | 234 | 0.707814 | 0.00001 | 17 | M | Middle Adult | D/2 | Hyksos | UV |
| A/II-n/13 Grab 1, Bestattung 3 | 282 | 0.707887 | 0.000009 | 37 | M | Young Adult | D/2 | Hyksos | UV |
| A/II-n/13 Grab 1, Bestattung 4 | 235 | 0.707784 | 0.000012 | 37 | --- | Adolescent | D/2 | Hyksos | UV |
| A/II-n/13 Grab 10 | 239 | 0.708015 | 0.000006 | 38 | M | Middle Adult | E/3 | 12th/13th Dynasty | UV |
| A/II-n/13 Grab 8, Bestattung 1 | 237 | 0.707744 | 0.000007 | 47 | M | Young Adult | D/3 | Hyksos | UV |
| A/II-n/13 Grab 8, Bestattung 2 | 238 | 0.70777 | 0.00001 | 16 | --- | Adolescent | D/3 | Hyksos | UV |
| A/II-n/15 Grab 2, Bestattung 2 | 241 | 0.707819 | 0.000008 | 47 | --- | Adolescent | F | 12th/13th Dynasty | UV |
| A/II-n/16 Grab 2 | 257 | 0.707857 | 0.000009 | 48 | F | Middle Adult | G | 12th/13th Dynasty | UV |
| A/II-n/19 Grab 4, Bestattung 2 | 242 | 0.707823 | 0.000009 | 37 | F | Young Adult | E/1 | Hyksos | UV |
| A/II-n/19 Grab 4, Bestattung 3 | 243 | 0.707795 | 0.000007 | 37 | --- | Adolescent | E/1 | Hyksos | UV |
| A/II-o/13 Grab 1, Bestattung 1 | 244 | 0.708228 | 0.000008 | 15 | F | Middle Adult | E/3 | 12th/13th Dynasty | UV |
| A/II-o/13 Grab 1, Bestattung 2 | 245 | 0.708447 | 0.00001 | 47 | F | Young Adult | E/3 | 12th/13th Dynasty | UV |
| A/II-o/20 Grab 4 | 246 | 0.707749 | 0.000006 | 47 | M | Middle Adult | E/1 | Hyksos | UV |
| A/II-o/20 Grab 5 | 247 | 0.707754 | 0.000008 | 37 | F | Young Adult | E/3-1 | --- | UV |
| A/II-o/21 Grab 3 | 248 | 0.707694 | 0.000007 | 47 | F | Young Adult | F-E/3 | 12th/13th Dynasty | UV |
| A/II-p/20 Grab 2 | 249 | 0.707747 | 0.000011 | 47 | F | Young Adult | E/2-1 | Hyksos | UV |
| A/II-p/21 Grab 12 (2) | 334 | 0.707678 | 0.000007 | 36 | I | Adult | F-E/3 | 12th/13th Dynasty | UV |
| A/II-p/21 Grab 15 | 253 | 0.708309 | 0.000008 | 47 | F | Middle Adult | D/3 | Hyksos | UV |
| A/II-p/21 Grab 3, Bestattung 1 | 304 | 0.707566 | 0.000007 | 28 | F | Old Adult | E/1-D/3 | Hyksos | UV |
| A/II-q/20 Grab 1 | 305 | 0.708338 | 0.000008 | 17 | F | Old Adult | E/1 | Hyksos | UV |
| A/II-r/18 Grab 1 | 255 | 0.708069 | 0.000009 | 17 | F | Middle/ Old Adult | F | 12th/13th Dynasty | UV |
| A/II-r/18 Grab 2 | 256 | 0.707998 | 0.000008 | 37 | F | Middle Adult | G | 12th/13th Dynasty | UV |
| F/I-i/21 Grab 30 | 329 | 0.70801 | 0.000009 | 47 | --- | --- | Ec | 12th/13th Dynasty | UV |
| F/I-i/21 Grab 34 | 330 | 0.707931 | 0.000006 | 37 | --- | --- | c | Hyksos | UV |
| F/I-j/22 Grab 30 | 331 | 0.707939 | 0.000008 | 27 | --- | --- | Ec | 12th/13th Dynasty | UV |
| F/I-j/22 Grab 31 | 259 | 0.708388 | 0.000009 | 27 | F? | Young Adult | c | Hyksos | MUV |
| F/I-j/23 Grab 13 | 260 | 0.707895 | 0.000007 | 27 | --- | --- | Ec | 12th/13th Dynasty | MUV |
| F/I-j/23(S) Grab 24 | 261 | 0.707808 | 0.000008 | 37 | --- | Adult | b/3-2 | 12th/13th Dynasty | MUV |
| F/I-k/22 Grab 19 | 262 | 0.707924 | 0.00001 | 27 | --- | Young Adult | a/1-2 | Hyksos | MUV |
